# Phytochemical and Biological Activities of Four Wild Medicinal Plants

**DOI:** 10.1155/2014/857363

**Published:** 2014-10-13

**Authors:** Anwar Ali Shad, Shabir Ahmad, Riaz Ullah, Naser M. AbdEl-Salam, H. Fouad, Najeeb Ur Rehman, Hidayat Hussain, Wajid Saeed

**Affiliations:** ^1^Agricultural Chemistry Department, The University of Agriculture, Peshawar, Khyber Pakhtunkhwa 25000, Pakistan; ^2^Department of Chemistry, Islamia College Peshawar, Khyber Pakhtunkhwa 25000, Pakistan; ^3^Department of Chemistry, Government College Ara Khel, FR Kohat, Khyber Pakhtunkhwa 26000, Pakistan; ^4^Riyadh Community College, King Saud University, Riyadh 11437, Saudi Arabia; ^5^Department of Biomedical Engineering, Faculty of Engineering, Helwan University, Helwan 11790, Egypt; ^6^UoN Chair of Oman's Medicinal Plants and Marine Natural Products, University of Nizwa, P.O. Box 33, Birkat Al Mauz, 616 Nizwa, Oman

## Abstract

The fruits of four wild plants, namely, *Capparis decidua, Ficus carica, Syzygium cumini*, and *Ziziphus jujuba*, are separately used as traditional dietary and remedial agents in remote areas of Khyber Pakhtunkhwa, Pakistan. The results of our study on these four plants revealed that the examined fruits were a valuable source of nutraceuticals and exhibited good level of antimicrobial activity. The fruits of these four investigated plants are promising source of polyphenols, flavonoids, alkaloids, terpenoids, and saponins. These four plants' fruits are good sources of iron, zinc, copper, manganese, selenium, and chromium. It was also observed that these fruits are potential source of antioxidant agent and the possible reason could be that these samples had good amount of phytochemicals. Hence, the proper propagation, conservation, and chemical investigation are recommended so that these fruits should be incorporated for the eradication of food and health related problems.

## 1. Introduction

The wild plants play significant role in the suppression of dietary and pathogen related ailments of native people since long before recorded history. Latest research investigation observed that the bioactive and antioxidant potentials of these plants are attributed to the presence of polyphenols, flavonoids, lignins, alkaloids, terpenoids, carotenoids, vitamins, and so forth [1–3]. They help in maintaining the nutritional quality and shelf life of foods by inhibiting lipid oxidation, minimizing rancidity, and removing toxic oxidative products [4–7]. Similarly, phenolic compounds play important role in antioxidant activity and resistance against pests and other species dissemination.

The emergent population coupled with poverty and natural disasters interrogates how the native people of Khyber Pakhtunkhwa are able to cope with the challenge of easy access to food and medicine. Despite the government's mammoth expenses on the livelihood of common people, the provision of balanced food and modern healthcare to rural people is still a far-reaching goal [8]. Hence, it is recommended that researchers should resort to forms of nutraceuticals mainly in the native plant species to overcome the constraints of human necessities. Phytochemicals and minerals ingredients are necessary for virtually all reactions to occur in the body [9–11]. While each has its own unique properties, they work synergistically to ensure reactions in the body occur appropriately [12].

Southern parts of Khyber Pakhtunkhwa are blessed with plenty of wild plants which have very significant role in the daily life of rural people. The main areas of this region include Kohat, Karak, Bannu, and Dera Ismail Khan. In this regard, a project was designed to investigate four different food plants (*Ficus carica*,* Syzygium cumini*,* Capparis decidua*, and* Ziziphus jujube*) that are separately used in cuisines either in fresh form or in semicooked form. It was also observed that some people of the subject areas use them for the eradication of various diseases. The botanical description of each examined plant is described briefly as follows.

*Ficus carica* L. is important species of Moraceae family which is locally known as Inzar in Pushto. It is widely grown throughout the temperate world, both for its fruit and as an ornamental. The fruits and leaves parts of this plant exhibited significant level of antioxidant and antimicrobial activity [13, 14]. The fruit has a laxative effect and the daily uses of dried fig enhanced the antioxidant capacity in plasma [15]. Similarly, Jamun (*Syzygium cumini* L. Skeels) belongs to Myrtaceae family and is considered the richest nutritional source. It contains flavonoids, tannins, triterpenoids, carotenoids, and sitosterols. The extracts of this plant exhibited various activities including cytotoxic, anti-inflammatory, anticancer, and antidiabetic activities [16].

The fruit of* Capparis decidua* Forssk. Edgew. (Capparidaceae) normally known as Khair is a wonderful source of alkaloids, terpenoids, glycosides, and some fatty acids. The plant has significant hypercholesterolemic, anti-inflammatory, analgesic, antidiabetic, antimicrobial, antiplaque, antihypertensive, anthelmintic, and purgative potential [17–21]. Likewise, Ber (*Ziziphus jujuba* Mill.) of family Rhamnaceae widely grows in southern parts of Khyber Pakhtunkhwa province of Pakistan. The fruits and seeds are used for antifungal, antibacterial, antiulcer, anti-inflammatory, sedative, antispastic, antifertility/contraception, hypotensive, and wound healing properties [22].

## 2. Materials and Methods

All the chemical studies including phytochemical screening, antimicrobial activity, antioxidant activity, and polyphenolic content of medicinal plants were conducted at Agricultural Chemistry Department, University of Agriculture, Peshawar, and Pharmacy Department, Peshawar University.

### 2.1. Plant Collection and Identification

The fresh fruit samples (*Ziziphus jujuba*,* Capparis decidua*,* Ficus carica*, and* Syzygium cumini*) were collected from different locations of Kohat, Karak, Bannu, and D. I. Khan-Khyber Pakhtunkhwa in 2008–2010. Each fruit along with aerial part of the fruit plant was identified by Mr. Shahid Farooq (Plant Taxonomist, PCSIR, Peshawar). A set of voucher specimens of each plant was deposited in the herbarium of PCSIR, Peshawar.

### 2.2. Sampling

About 3–5 kg samples of each plant fruit material were collected from their natural habitat from 4-5 different sites. About 1-2 kg representative sample of each plant was thoroughly mixed and immediately washed of dust, impurities, and other adhering materials using tap water. The fruit sample was then dried at room temperature under shade for 4-5 days or until the fruits were completely dried. The dried samples were first ground into small pieces by use of mortar and pestle and then into fine powder by use of electrical grinder. These powdered samples were sealed in plastic bags and stored in refrigerator until they were analyzed.

### 2.3. Extraction

The powder plants samples were macerated in aqueous methanol for primary extraction. The filtrates were then evaporated using rotary evaporator.

### 2.4. Phytochemical Screening

Phytochemical screening of the subject samples for the qualitative identification of tannins, saponins, terpenoids, steroids, and flavonoids was done by various analytical techniques. Standard procedure was used for screening of the constituents as described by [23–25].

#### 2.4.1. Determination of Tannins

About 0.5 g dry powder of each sample was boiled in 20 mL of water in a test tube and then filtered. Few drops of 0.1% ferric chloride solution were added and noticed for brownish green or blue-black coloration [24].

#### 2.4.2. Determination of Saponins

About 2 g of the powder sample was boiled in 20 mL of distilled water on a water bath and filtered. 10 mL filtrate was mixed with 5 mL of distilled water and shaken vigorously for a stable persistent froth. Then, the froth was mixed with 3 drops of olive oil and shaken vigorously, till emulsion formed [23].

#### 2.4.3. Determination of Flavonoids

Approximately 5 mL of the dilute ammonia solution was added to a portion of the aqueous filtrate of each plant extract followed by addition of concentrated H_2_SO_4_. A yellow coloration was observed in each extract indicating the presence of flavonoids. The yellow coloration disappeared on standing [24].

#### 2.4.4. Determination of Terpenoids

About 5 mL of each plant extract was mixed with 2 mL of chloroform and after mixing about 3 mL concentrated H_2_SO_4_ was carefully added to form a layer. A reddish brown coloration of the inner face will be formed indicating the presence of terpenoids [25].

#### 2.4.5. Determination of Steroids

About 20 g of each plant sample was soaked in ethanol in a conical flask. The sample containing ethanol was warmly heated in water bath and then filtered. In this process the ethanol fraction was extracted and then the alcohol portion was evaporated from the filtrate by placing it in water bath in a large size watch glass. The solid sample left behind was dissolved in chloroform. Now acetic anhydride 4.5 mL and sulfuric acid 0.5 mL were added. During the experiment the color of the extract changed from violet to green, which was indicating the presence of steroids [23].

#### 2.4.6. Total Polyphenols Determination

Total polyphenolic content of the crude fractions of subject plants was evaluated through the FCR method in which the gallic acid was used as standard. 100 *μ*l of each plant sample was taken and subsequently 900 *μ*l of distilled water was added to the samples, after which 0.5 mL of FCR was added to the mixture. 20% of the Na_2_CO_3_ solution was made. From this solution 1.5 mL was taken and added into the above mixture (sample + water + FCR). The whole mixture was placed in volumetric flask on water bath and heated for about 2 hours. After the cooling of the mixture, UV-vis spectrophotometer was employed to measure the absorbance at the wavelength of 720 nm. Total polyphenolic contents in samples were assessed by using gallic acid equivalent (GAE) as standard [26].

#### 2.4.7. Total Flavonoid Contents Determination

Total flavonoid content of the crude fraction of each of the samples was evaluated through the calorimetric method in which Quercetin was taken as standard. One mL of each sample was taken and 4 mL of distilled water was added. To this mixture sodium nitrate and Alcl_3_ were added at the concentration mixture of 300 *μ*l. The total volume was then incubated for 5 minutes followed by the addition of 2 mL of NaOH; the volume was increased up to 10 mL. UV-vis spectrophotometer was used for absorbing the wavelength set at 510 nm and total flavones were then expressed as Quercetin equivalents in mg/g of dry samples.

### 2.5. Antioxidant Activity

#### 2.5.1. DPPH Radical Scavenging Assay

The powdered material of the subject plants was extracted with methanol for 36–48 h. The solvent after extraction of each sample was evaporated at low temperature under reduced pressure in rotary vacuum evaporator to obtain crude extracts.

Antioxidant activity was determined by using the 2,2-diphenyl-1-picrylhydrazyl (DPPH) radical scavenging assay as described by [27–30]. DPPH (1 mL, 0.3 mM) solution in ethanol was added to 2.5 mL (100 mg mL^−1^) of methanol extract and mixture was allowed to react for 30 min at room temperature and absorbance was recorded using UV visible spectrophotometer at 518 nm. HPLC grade Rutin hydrate (95%, 1 mM) was used as standard, while absolute ethanol was used as negative control. The experiment was repeated in triplicate, using fresh plant sampled each time, and the average absorbance values were converted to percentage antioxidant activity.

### 2.6. Antibacterial and Antifungal Activities

Bacterial and fungal strains tests were performed on three bacteria and two fungi reference strains. Bacterial strains were* Escherichia coli*,* Bacillus subtilis*, and* Staphylococcus aureus* while fungal strains include* Trichophyton longifusus* (clinical isolate) and* Candida albicans*. They were maintained on agar slant at 48°C. The strains were activated at 378°C for 24 hr on nutrient agar or Sabouraud glucose agar (SGA), respectively, for bacteria and for fungi, prior to any screening [31].

### 2.7. Minerals Assay

Dried and ground leaves samples were wet digested for minerals as stated by [32] in 1 : 1 mixture of HNO_3_-HClO_4_ by gradually increasing the temperature up to 300°C. Trace elements such as iron (Fe), copper (Cu), zinc (Zn), manganese (Mn), and selenium (Se) were determined using double beam atomic absorption spectrophotometer Perkins Elmer Model 2380.

## 3. Results and Discussion

In the present study, four wild plants found in southern parts of Khyber Pakhtunkhwa were investigated for their dietary and pharmacological potential. These plants (*Capparis decidua*,* Ficus carica*,* Syzygium cumini*, and* Ziziphus jujuba*) were investigated for secondary metabolites using phytochemical screening, biochemical compounds and mineral content, antimicrobial potential, and antioxidant activity. In the present study, selected ethnobotanical information is displayed in Table 1. This information was obtained during the collection time from the native populace. The study of literature also confirmed its ethnobotanical potential [18–20]. These plants are separately used by local dwellers. Although various parts of the examined plants are used for different purposes, the fruit parts are very popular among the people. The fruit part of* Capparis decidua* plant is known as karir or kary which is edible and also used in various forms of processed food like pickles and making sweet food products. The flowers are used as a vegetable and commonly consumed with loaf and meat. The* Ficus carica* locally known as Inzar is widely distributed in different parts of Khyber Pakhtunkhwa. Fruit and latex are routinely used for dietary and medicinal purposes. The fruit part is used in both fresh and dried forms and it is also used in making sweet food products like cake and bakery making [33, 34].

*Syzygium cumini* locally known as Jaman is very famous for its dietary and medicinal values. The leaves, fruit, seeds, and bark are very effective against anti-inflammatory activity, asthma, diarrhea, fever, diabetes, and other common ailments [35]. The fruits are consumed in different parts of Pakistan as fresh raw fruit as such or in salad or processed in jams, squash, and so forth. Similarly the* Ziziphus jujuba* plant is commonly known as baira and it is one of the widely grown plants of southern parts of Khyber Pakhtunkhwa. The fruit is liked by people of all ages and used both in dried and in fresh forms. It is also used in sweet food products making and used for the eradication of bronchitis, diarrhoea, dysentery, cold, and coughs.

Phytochemicals are natural bioactive compound widely distributed in plants, animals, microbes, and other forms of life. The principal examples of natural products including alkaloids, terpenoids, steroids, polyphenols, and flavonoids have rational uses and are found in varying amounts in different species. Their presence in the food chains has significant role which is to work with nutrients and dietary fibre to protect against disease [36–38]. Nevertheless, their presence in foods has traditionally been regarded as antinutritional factor [39] and in some cases has limited their use due to their bitter taste [40, 41].

In the present study, the preliminary phytochemical screening of the subject plants reveals that they are good sources of natural products. The results concluded that the studied plants contained appreciable amount of tannins, flavonoids, steroids, alkaloids, and saponins; however terpenoids content was almost negligible (Table 2). The results regarding flavonoids and tannins were very promising in the investigated plants. The preliminary phytochemical screening of* Syzygium cumini* and* Ziziphus jujuba* showed promising results for the presence of saponins, tannins, terpenoids, and flavonoids while the examined plants revealed the least level of steroids concentration in the crude aqueous methanolic extract. Similarly, Borhade [42] reported that flavonoids, tannins, steroids, and saponins are the main constituents in the* Syzygium cumini* plant. It is confirmed from the literature that larger amount of flavonoids might be responsible for their healing effect against pathogenic microorganisms [43]. Similarly, saponins concentration is appreciable in* Capparis decidua* and* Ficus carica* and has the property of binding with cholesterol, bitterness, and hemolytic activity in aqueous solution [44].

In the present study all the four subject plants were investigated for total polyphenols and flavonoids content and their results are oscillated in Table 3. Phenols are the key component of many biocompounds like salicylic acid (aspirin) and recent literature studies reported that thousands of novel and variant phenolic compounds were identified and discovered in different biological sources. They are the key unit in all the foods we normally consume. The primary role of their presence in our diets is acting as antioxidant, antiseptic, and anti-inflammatory and for the eradication of other different human aliments. They also have nuisance effect when applied directly to the skin.

Flavonoids are hydroxylated phenolic substances, responsible for their therapeutic potency against wide array of microorganisms, probably due to their ability to complex with extracellular and soluble proteins and to complex with the bacterial cell wall. Flavonoids and other phenolic compounds are potent water soluble antioxidants and free radical scavengers, which prevent oxidative cell damage and have strong anticancer activity [45, 46].

The aim of this test was to evaluate the absorbing capacity of subject plants in two different fractions (hexane and methanol) by using Folin-Ciocalteu reagent. Gallic acid was used as standard to evaluate the results obtained. The results showed total phenolic content and total flavonoid contents were found in promising amount in all the studied plants ranging from 4 to 31 mg GAE/g and from 3 to 43 mg Quercetin/g, respectively. The maximum content of total phenolic compound was observed in methanolic fraction of* Syzygium cumini* (31.2 ± 3.15 mg GAE/g) followed by* Capparis decidua* (23.2 ± 3.15 mg GAE/g) and low level of the content was found in hexane fraction of* Syzygium cumini* (4.2 ± 3.28 mg GAE/g). Similarly, the flavonoid content of the* Capparis decidua* was maximum in methanolic extract (43.46 ± 12.52 mg Quercetin/g) and low level of the content was found in hexane fraction of* Ziziphus jujuba* (3.25 ± 1.35 mg Quercetin/g).

Research has confirmed that subject plants possess total phenolic and flavonoids contents which are of enormous benefits for mankind, thus ratifying our findings. The antioxidant support we get from investigated plants is largely due to their phenolic compounds that have been shown to help protect us against unwanted oxygen damage to our cells, blood vessels, and organ systems [47]. Antioxidant compounds in food play an important role as a health protecting factor. Scientific evidence suggests that antioxidants reduce the risk for chronic diseases including cancer and heart disease. It is increasingly being realized that many of today's diseases are due to the “oxidative stress” that results from an imbalance between formation and neutralization of prooxidants. Oxidative stress is initiated by free radicals; hence, all human beings protect themselves against free radical damage somehow by antioxidant supplements, which are vital to combat oxidative damage. It is also called free radical scavenging activity because of its ability to scavenge free radicals. Free radical generation is directly related with oxidation in foods and biological systems. Therefore, the search for potential free radical scavenging agent is imperative and indispensable.

Table 4 displays the percent antioxidant activity of studied plants using 2,2-diphenyl-1-picrylhydrazyl (DPPH) radical. In this study, three different concentrations were selected (0.25, 0.50, and 1.00 mg). It was observed that highest antioxidant activity was observed in 1.00 mg content of* Syzygium cumini* (63.3%) followed by* Ziziphus jujuba* (47.3%),* Capparis decidua* (15.5%), and* Ficus carica* (10.9%), respectively. However, slightly different trends were observed in analyzing the 0.25 and 0.5 mg content of the examined plants. In this case,* Ficus carica* exhibited maximum antioxidant activity at 0.5 mg level (27.3%) followed by 0.25 mg level (19.5%).

In the present research study, methanolic crude extracts of* Capparis decidua*,* Ficus carica*,* Syzygium cumini*, and* Ziziphus jujuba* were evaluated for antimicrobial potential (Table 5). They were investigated against three different bacterial strains (*E. coli*,* P. aeruginosa*, and* S. typhi*) and two different fungal strands including* Trichophyton longifusus* and* Candida albicans*. Minimum inhibitory concentrations of the effective extracts were estimated by agar dilution method. The results were compared with that of standard drug.

Similarly,* P. aeruginosa* is a frequent cause of nosocomial infections such as pneumonia, urinary tract infections (UTIs), and bacteremia [48].* Salmonella typhi* is the main food poisoning agent and typhoid fever that is a major public health problem worldwide.

Likewise, the pathogenic fungi* Trichophyton longifusus* are responsible for the infections of dermatophytosis in the hair, skin, and nails of humans due to their ability to utilize keratin as a source of nourishment. Another fungal strain* Candida albicans* is responsible for the candidiasis which affects various body parts including ear, mouth, throat, skin, scalp, fingers, toes, nails, bronchi, lungs, and intestine gastrointestinal tract. Factors predisposing people to candidiasis include AIDS, burn patients, young individual, pregnancy, oral birth control, high fruit diets, steroids, antibiotic therapy, immune suppressants, cancer treatments, heart surgery, genetic deficiency, and endocrine deficiency diabetes.

Results of the study divulged that* Capparis decidua*,* Ficus carica*,* Syzygium cumini*, and* Ziziphus jujuba* had potent antibacterial agent against* E. coli*,* S. typhi*, and* P. aeruginosa*. It was observed that crude methanolic fraction of* Ficus carica*,* Syzygium cumini*, and* Ziziphus jujuba* exhibited good level of activity against all investigated strains. Similarly, the* Ziziphus jujuba* is effective against* E. coli* showing MIC value of 0.60 ± 0.1 and is least effective against* P. aeruginosa*.* E. coli* is one of the most frequent agents of many bacterial infections. The major diseases caused by this strain are cholecystitis, bacteremia, cholangitis, urinary tract infection, diarrhoea, neonatal meningitis, and pneumonia [49]. Similarly,* Ficus carica* and* Syzygium cumini* exhibited activity against both bacterial and fungal strains. The possible reasons of this activity might be due to the presence of phytochemicals. The literature studies reveal that these natural products have significant role in the biological and pharmacological activity.

Likewise, the investigated plants were also analysed for the elemental composition. Mean values of the data reported in Table 6 showed that manganese and iron are found in maximum concentration followed by zinc whereas Cr is found in minimum level. Manganese showed a concentration ranging from 0.53 to 12 mg 100^−1^ g. It has been reported previously that chromium nutrition leads to decreased requirement for insulin and improved blood lipid profile [50]. It is observed that zinc is present with promising concentration of 1.3 mg 100^−1^ g in* Capparis decidua*. Nevertheless, it was found that the subject plants are good sources of these minerals and they might be beneficial in the eradication of certain diseases.

## 4. Conclusion

The current study showed that the investigated plants' fruits of all four plants are good sources of iron, zinc, copper, manganese, selenium, and chromium. It was also observed that these fruits are potential source of antioxidant agent and the possible reason could be that these samples had good amount of phytochemicals. Hence, the proper propagation, conservation, and chemical investigation are recommended so that these fruits should be incorporated for the eradication of food and health related problems.

## Figures and Tables

**Table 1:** Ethnobotanical information of selected species of subject area used as food and medicine.

S. number	Botanical name	Family	Local name	Life form	Parts used	Mode/form of consumption	Perceived traditional ethnobotanical use
1	*Capparis decidua* Edgew	Capparidaceae	Karir	Shrub	Flower and fruit	Raw, dried, semicooked, and used in making pickles	Dietary and medicinal

2	*Ficus carica* Forssk	Moraceae	Inzar (Injeer)	Shrub/small tree	Milky latex and fruits	Raw, dried, decoction, eaten fresh, and used in salad, jam, marmalade, and bakery products	Dietary and medicinal

3	*Syzygium cumini* L.	Myrtaceae	Jaman	Tree	Fruits, seeds, leaves, and bark	Raw and extracts and used in salad, jam, jellies, refreshing drinks, and other sweet food products	Dietary and medicinal (diabetes)

4	*Ziziphus jujuba* Mill.	Rhamnaceae	Baira	Tree	Leaves, bark, and fruits	Raw, decoction, and dried	Dietary and medicinal

**Table 2:** Phytochemical screening of methanolic extract of investigated samples.

Names	*Capparis decidua *	*Ficus carica *	*Syzygium cumini *	*Ziziphus jujuba *
Tannins	++	++	++	++
Flavonoids	++	++	++	++
Alkaloids	++	++	++	++
Phenols	++	++	++	++
Saponins	−	++	++	++
Steroids	++	++	++	++

++: present and −: absent.

**Table 3:** The phytochemical constituents (flavonoid extract and total phenolic content) of selected plants form the studied area.

Samples/extracts	Total phenolic contents∗ ± SD		Total flavonoid∗∗ Content ± SD	
	Hexane extract	Me.OH extract	Hexane extract	Me.OH extract
*Capparis decidua* Edgew	11.15 ± 4.32	23.2 ± 3.15	26.60 ± 12.28	43.46 ± 12.52
*Ficus carica* Forssk	5.95 ± 2.47	16.95 ± 3.23	26.95 ± 7.17	36.60 ± 12.28
*Syzygium cumini* L.	4.2 ± 3.28	31.2 ± 3.15	13.2 ± 3.15	25.2 ± 3.15
*Ziziphus jujuba* Mill	5.78 ± 2.17	21.74 ± 4.35	3.25 ± 1.35	15.66 ± 3.87

∗mg GAE/g of extract and ∗∗mg Quercetin equivalent/g of extract.

**Table 4:** Antioxidant activity (%) of the studied plants found from various regions of KPK.

Plant species	Concentration (mg)		
	0.25	0.50	1.00
*Capparis decidua *	9.1	12.3	15.5
*Ficus carica* Forssk	27.3	19.1	10.9
*Syzygium cumini* L.	35.0	49.2	63.3
*Ziziphus jujuba *	34.9	41.1	47.3

**Table 5:** Antimicrobial (antifungal and antibacterial) assay at 5 mg/mL of methanolic crude extracts of selected plants (average value ± SD, *µ*g/mL).

Fractions	*EC*	*PA*	*ST*	*TL*	*CA*
*Capparis decidua *	17.7 ± 6.58	11.16 ± 2.05	0.96 ± 0.1	0.82 ± 0.28	1.11 ± 0.50
*Ficus carica *	0.74 ± 0.15	0.67 ± 0.24	0.12 ± 0.03	1.17 ± 0.30	0.5 ± 0.2
*Syzygium cumini *	0.73 ± 0.20	10.0 ± 1.64	0.54 ± 0.23	0.46 ± 0.15	0.46 ± 0.01
*Ziziphus jujuba *	0.60 ± 0.1	3.40 ± 0.49	0.93 ± 0.16	0.85 ± 0.07	—
**Cipro**/**Ampho.B**	0.06	1.5	0.25	0.1	0.5

*EC: E. coli; PA: P. aeruginosa; ST: S. typhi, TL: Trichophyton longifusus; CA: Candida albicans*. Cipro and Ampho.B (standard drug), SD: standard deviation.

**Table 6:** Mineral composition of investigated plants selected from southern region of KPK (mg 100^−1^ g).

Elements	*Capparis decidua *	*Ficus carica *	*Syzygium cumini *	*Ziziphus jujuba *
Iron	6.13 ± 0.15	9.07 ± 2.45	1.90 ± 0.30	7.40 ± 0.20
Zinc	1.30 ± 0.10	3.00 ± 1.50	1.17 ± 0.58	2.57 ± 0.30
Copper	2.50 ± 0.10	4.63 ± 1.52	1.37 ± 0.13	1.34 ± 0.05
Manganese	12.30 ± 0.20	7.12 ± 2.10	6.33 ± 0.58	0.53 ± 0.08
Selenium	0.25 ± 0.08	0.39 ± 0.58	0.35 ± 0.05	0.41 ± 0.22
Chromium	0.46 ± 0.11	1.57 ± 0.47	ND	ND

Values are for triplicate determination (mean ± SD).
